# The human long noncoding RNAs CoroMarker, MALAT1, CDR1as, and LINC00460 in whole blood of individuals after controlled short-term exposure with ultrafine metal fume particles at workplace conditions, and in human macrophages in vitro

**DOI:** 10.1186/s12995-022-00356-0

**Published:** 2022-08-01

**Authors:** Theresa Scheurer, Jan Steffens, Agnieszka Markert, Miriam Du Marchie Sarvaas, Christoph Roderburg, Lothar Rink, Frank Tacke, Tom Luedde, Thomas Kraus, Ralf Baumann

**Affiliations:** 1 Institute for Occupational, Social and Environmental Medicine, Medical Faculty, University Hospital RWTH Aachen University, Pauwelsstr. 30, 52074 Aachen, Germany; 2 Institute for Translational Medicine (ITM), Medical School Hamburg (MSH) – Am Kaiserkai 1, 20457 Hamburg, Germany; 3 Department of Medicine III, Medical Faculty, University Hospital RWTH Aachen University, Pauwelsstr. 30, 52074 Aachen, Germany; 4 Department of Hepatology and Gastroenterology, Charité - Universitätsmedizin Berlin, Augustenburger Platz 1, 13353 Berlin, Germany; 5 Clinic for Gastroenterology, Hepatology and Infectious Diseases, University Hospital Düsseldorf, Moorenstr. 5, 40225 Duesseldorf, Germany; 6 Institute of Immunology, Medical Faculty, University Hospital RWTH Aachen University, Pauwelsstr. 30, 52074 Aachen, Germany

**Keywords:** LncRNA, Occupational health, Zinc/copper (Zn/cu) metal fume exposure, Nanotoxicology, Macrophages

## Abstract

**Background:**

Short-term inhalation of occupationally relevant ultrafine zinc/copper (Zn/Cu) containing welding fumes has been shown to induce subclinical systemic inflammation, associated with an elevated risk for cardiovascular diseases. The involvement of noncoding RNAs (lncRNAs) in this setting is currently unknown. However, lncRNAs have been reported to fulfill essential roles in, e.g., cardiovascular diseases, inflammation, infectious diseases, and pollution-related lung disorders.

**Methods:**

In this study, the specific lncRNAs levels of the 4 lncRNAs CoroMarker, MALAT1, CDR1as and LINC00460 were determined by RT-qPCR in THP-1 macrophages exposed to Zn/Cu metal fume suspensions for 1, 2, and 4 hours in vitro. Furthermore, 14 subjects were exposed to Zn/Cu containing welding fumes (at 2.5 mg/m^3^) for 6 hours. Before, 6, 10, and 29 hours after exposure start, whole blood cell lncRNAs levels were determined by RT-qPCR.

**Results:**

In THP-1 macrophages, we observed a 2.3-fold increase of CDR1as at 1 h (Wilcoxon *p* = 0.03), a non-significant increase of CoroMarker at 1 h, and an increase of LINC00460 at 2 h (*p* = 0.03) and at 4 h (*p* = 0.06). In whole blood cells, we determined a non-significant upregulation of CDR1as at 6 h (*p* = 0.2), a significant downregulation of CoroMarker at 6 h (*p* = 0.04), and a significant upregulation of LINC00460 levels at 10 h (*p* = 0.04) and 29 h (*p* = 0.04). MALAT-1 remained unchanged in both settings.

**Conclusion:**

The orientation of regulation of the lncRNAs is (except for CoroMarker) similar in the in vitro and in vivo experiments and in line with their described functions. Therefore, these results, e.g. the upregulation of the potential risk marker for cardiovascular diseases, CDR1as, contribute to understanding the underlying mechanisms of Zn/Cu-induced subclinical inflammation in metal workers.

## Background

Many metal workers are exposed to welding fumes, mainly consisting of agglomerated metal oxide particles primarily built up of easily respirable nanoparticles [1]. Exposure to welding fumes is associated with inflammatory responses in the airways and systemic subclinical inflammatory effects. In the long run, it may increase the risk for pulmonary diseases, e.g. COPD, occupational asthma, pneumonia, and chronic bronchitis, and for systemic diseases such as cardiovascular diseases [2–5].

Recently, the heavy metals zinc (Zn) and copper (Cu) have become more common in modern joining technology, especially in the automotive industry [6]. Controlled short-term exposure with zinc oxide fume resulted in increased levels of TNF, IL-6, and IL-8 in bronchoalveolar lavage (BAL) fluid of exposed welders [7] and/or exposed healthy subjects [8].

Furthermore, a controlled 6 hour-exposure to zinc- and copper-containing (Zn/Cu) welding fume caused significant increases of the acute-phase reactants C-reactive protein (CRP) and serum amyloid A (SAA) in nasal mucosal lining fluid [9]. These local inflammatory responses in the upper airways preceded and accompanied significant systemic inflammatory responses characterized by a highly significant early increase of the acute phase mediator IL-6 at 10 h and significant increases of CRP and SAA at 29 h after exposure start [9–12]. Comparable systemic CRP-increases were caused by welding fumes containing only zinc or only copper [12]. Repeated controlled exposure of healthy male subjects to zinc- and copper-containing welding fumes for 6  hours on 4 consecutive days each showed a persistent increase of systemic inflammatory markers such as CRP [13]. As IL-6, CRP and SAA represent independent risk markers for future cardiovascular events, these data may be crucial for long-term metal workers and welders concerning their cardiovascular health. Consistently, a long-term study showed that long-term welders have a higher risk for cardiovascular diseases [4]. After inhalation of ZnO, the effects on the cardiovascular system did not show measurable electrocardiographic changes, suggesting the changes happen at the cellular level [14].

This adds up to the increased risk for the above-mentioned airway diseases. For example, the severity, duration, and frequency of respiratory infections are increased among welders [2]. Alveolar macrophages play a significant role in lung inflammation due to their essential role in the defense against pollutants and pathogenic microbes. In an in vitro study on the effect of nanoparticles on human macrophages, copper- and zinc-based nanoparticles proved to be the most toxic among 24 different metals [15]. Moreover, exposure of macrophages to ZnO nanoparticles decreased lysosomal stability and increased cell death [16].

Taken together, these studies indicate that it is important to explore further the inflammatory and toxicological reactions to nano-sized zinc and copper particles.

In the past few years, research has shown that long noncoding RNAs (lncRNAs), which are over 200 nucleotides in length and lack protein-coding capacity, fulfill essential roles in, e.g., cardiovascular diseases, infectious diseases, pollution-related lung disorders, (nano-)toxicology, and inflammation [17]. Due to reported links to IL-6, inflammation, cardiovascular diseases, and/or toxicology, we selected the following four lncRNAs: CDR1as [18, 19], CoroMarker [20, 21], LINC00460 [22], and MALAT-1 [23–25] as potential biomarkers for the effects of Zn/Cu particles.

We investigated the expression levels of these four lncRNAs in two different settings, first in THP-1 macrophages exposed to Zn/Cu metal fume suspensions for 1, 2, and 4 hours in vitro; secondly, in the white blood cells derived from healthy, non-smoking male subjects after controlled exposure with ultrafine Zn/Cu containing welding fume particles (at 2.5 mg/m^3^) for 6 hours.

## Materials and methods

### Cell culture experiments

The in vitro suspension cultures of THP-1 monocytes and their differentiation to macrophages followed standard procedures (Appendix 1). Exposures to Cu/Zn metal fume (welding fume) suspension (Appendix 2) for 1 hour, 2 hours, and 4 hours took place in a starvation medium (Appendix 1) (For logistical reasons, at 2 hours, 2 of the 6 experiments were harvested not at 120 min but at 130 min). The medium was removed for cell harvesting, and 700 μl QIAzol (Qiagen, Hilden, Germany) was added. After 5 min incubation, the lysed cells were transferred into a 2 ml reaction vessel (Sarstedt AG & Co. KG, Nümbrecht, Germany) and stored at − 80 °C.

### Design of the welding fume exposure study

The Ethics Committee approved all study protocols of the Medical Faculty of the RWTH Aachen University (EK 195/14). Blood was taken and analyzed from 14 healthy male non-smokers after written consent who had normal body plethysmography, had no medical preconditions such as atopy, asthma, lung, or cardiac disease and had not previously been exposed to welding fume. The study group was exposed for 6 h to welding fumes mainly consisting of copper and zinc particles under controlled conditions as previously described [10]. Each individual cycled on a bicycle ergometer at 80 W for 10 min every hour inside the exposure laboratory to simulate physical work [9–12, 26, 27]. Blood tubes were taken before exposure and 6, 10, and 29 hours after the initiation of exposure. After centrifugation, the cellular layer (buffy coat) was separated, aliquoted in 200 μl portions, and stored at − 80 °C.

### Spirometry during the welding fume exposure study

Lung function was measured before and after exposure using body plethysmography, including spirometric and impulse oscillometric measurements [9–11].

### Technical generation of the welding fume and controlled exposure

The controlled exposures to welding fume particles took place in the ‘Aachen Workplace Simulation Laboratory’ (AWSL), as described previously [26, 28]. A brief description and the technical details for the welding fume generation can be found in Appendix 2.

### Extraction of total RNA and conversion to complementary DNA

Directly before the total RNA extraction, blood sample aliquots were thawed on ice and liquefied with 100 μl PBS containing 2% RNase inhibitor. Like the cell culture samples, the samples were further processed using the miRNeasy Mini kit (ID 217004, Qiagen, Hilden, Germany) following the manufacturer’s manual. Due to the large volume, the sample volume was divided into two portions into two tubes and processed separately on ice. The final RNA-eluate was aliquoted and frozen at − 80 °C. The High Capacity cDNA Reverse Transcription Kit (ID 4374966, Thermo Fisher Scientific, Massachusetts, USA) was used to convert total RNA to complementary DNA (cDNA) following the manufacturer’s manual. Afterward, the cDNA samples were aliquoted and stored at − 20 °C.

### RT-qPCR measurements

In contrast to the other 3 lncRNAs, CDR1as exists in a circular form called circRNA-7 s [29] but is nevertheless detectable through primer-based real-time polymerase chain reaction (RT-qPCR) [30]. For the PCR, 7.5 μl reaction mix consisting of 5.0 μl MasterMix (SensiFAST SYBR Lo-ROX Kit #BIO-94050 (Bioline (Aust) Pty Ltd., Alexandria, Australia)), 0.5 μl Sense, and 0.5 μl Antisense lncRNA-Primer (Primer pairs [18, 21, 31–33] (Eurofins Scientific SE, Ebersberg, Germany)) and 1.5 μl RNase-free water was pipetted in each well, followed by 2.5 μl cDNA (1:20 diluted with 0.1xTE buffer). Before PCR start, the plate was briefly centrifuged to eliminate potential air bubbles.

The PCR was performed using a QuantStudio 5 Real-Time PCR System (Thermo Fisher Scientific, Massachusetts, USA) using a 2 min hot start at 95 °C, followed by 40 cycles consisting of 5 s at 95 °C and 20 s at 62 °C. The CT values were exported from the QuantStudio 5 software and analyzed using statistical software.

### Statistics

Statistical analyses were performed using MedCalc software (MedCalc Software, Oostende, Belgium) and GraphPad Prism software (Graph Pad, San Diego, CA, USA). To examine differences in the lncRNA expression levels between THP-1 macrophages cultured with or without welding fume suspension at 1, 2, and 4 hours, the Mann-Whitney test was applied, as the data were not normally distributed. To examine whole blood leucocyte lncRNA level differences between points on the metal fume challenge curve, Friedman analysis of variance (ANOVA) was used. If a significant overall difference was found, posthoc analysis was performed using the method given by Conover [34] to identify time points showing significantly different levels compared to the baseline. In parallel, the time points were also investigated statistically using the Wilcoxon test. These calculations were done on the level of dCT values and were confirmed on the level of ddCT values. A two-tailed *P*-value of *P* ≤ 0.05 was judged significant.

## Results

### Characteristics of the welding fume used in the controlled short-term human exposure study

The copper and zinc-containing AluBronze welding fume used for the controlled 6 h-exposure study has been described before in detail [10]. In brief, a total fume mass concentration of PM_10_ = 2.5 mg/m^3^ was used with the main constituents zinc [1.43 mg/m^3^ (57% of total fume mass concentration)] and copper [0.375 mg/m^3^ (15%)], as well as traces of aluminum [0.032 mg/m^3^ (1.3%)]. A recent scanning electron microscope (SEM) analysis of the welding fume particles in the exposure room showed spherically shaped particles in different sizes between 50 nm and 300 nm and independent sharper, needle- or drop-like particles [9]. The welding fume for the cell culture experiments in vitro had comparable target concentrations (tc) for copper [tc: 0.4 mg/m^3^] and zinc [tc: 1.5 mg/m^3^] but lacked the aluminum traces and was produced as previously described [26].

### Gene expression levels of four selected long noncoding RNAs in macrophages exposed to cu and Zn containing metal fume particle suspension

#### CDR1as expression levels

**Fig. 1:**
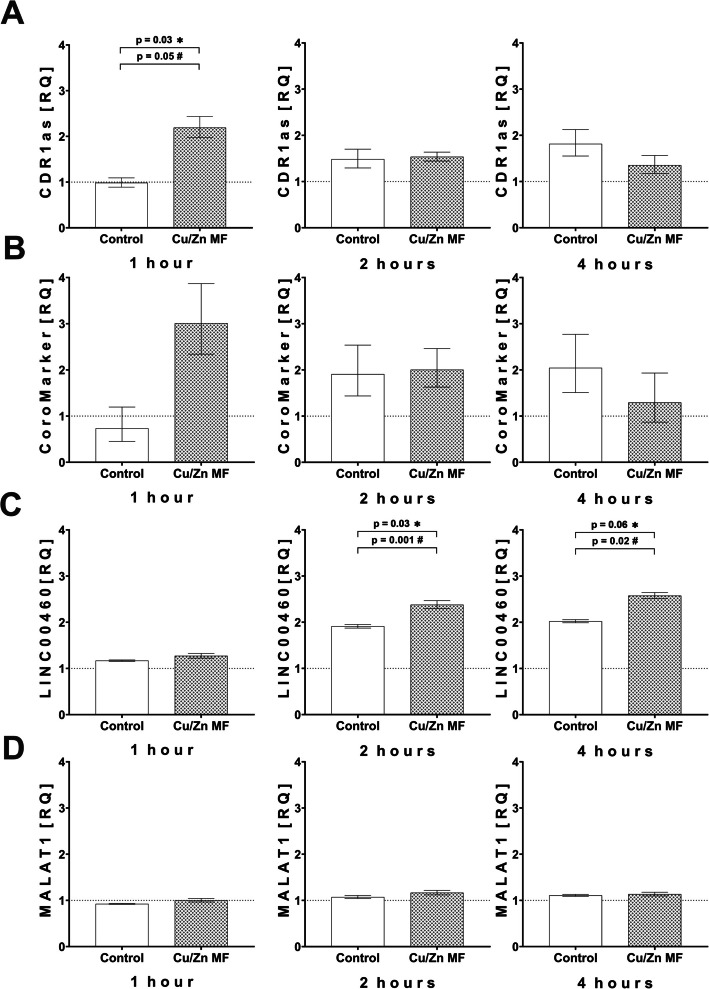
Expression levels of CDR1as (**A**), CoroMarker (**B**), LINC00460 (**C**), and MALAT1 (**D**) in Cu/Zn fume exposed macrophages. Time course of macrophage lncRNA levels after 1 h, 2 h, and 4 h exposure with 2 μg/ml Cu/Zn metal fume suspension  or control  . The data were measured by RT-qPCR normalized to GAPDH and are depicted as relative quantification (RQ) compared to baseline. Values presented are means (RQ_min_; RQ_max_; *n* = 6). ✱ Wilcoxon signed-rank test; # paired t test.

In THP-1 macrophages exposed to Zn/Cu metal fume suspensions for 1, 2, and 4 hours in vitro, lncRNA CDR1as levels increased 2.3-fold at 1 h (Wilcoxon *p* = 0.03; paired t-test *p* = 0.05) compared to the non-exposed control. The CDR1as levels did not show significant differences compared to control at 2 h and at 4 h (Fig. 1A).

#### CoroMarker expression levels

In THP-1 macrophages exposed to Zn/Cu metal fume suspensions in vitro, lncRNA CoroMarker levels increased non-significantly at 1 h (paired t-test *p* = 0.10) compared to the non-exposed control and returned to control levels at 2 h (Fig. 1B). Interestingly, the CoroMarker levels in the unexposed control rose clearly at 2 h and stayed elevated at 4 h.

#### MALAT1 expression levels

The lncRNA MALAT1 levels remained stable in THP-1 macrophages exposed to Zn/Cu metal fume suspensions in vitro (Fig. 1D).

#### LINC00460 expression levels

In THP-1 macrophages exposed to Zn/Cu metal fume suspensions in vitro, lncRNA LINC00460 levels remained unchanged at 1 h. They increased 1.4-fold at 2 h (Wilcoxon *p* = 0.03; paired t-test *p* = 0.001) and 1.5-fold at 4 h (Wilcoxon *p* = 0.06; paired t-test *p* = 0.02) compared to the non-exposed control, which showed higher levels at 2 h and 4 h compared to 1 h (Fig. 1C)).

### Kinetics of four selected long noncoding RNAs in whole blood cells of exposed individuals

The kinetics of the human long noncoding RNAs LINC00460, CoroMarker, MALAT1, and CDR1as were also investigated in whole blood leucocytes of individuals at 6 h, 10 h, and 29 h after the start of a controlled 6 h-exposure with the occupationally relevant Cu/Zn welding fume (at 2.5 mg/m^3^) containing ultrafine metal fume particles. The levels of the four lncRNAs were determined by RT-qPCR and normalized to the gene expression levels of GAPDH.

#### LINC00460 expression levels

**Fig. 2:**
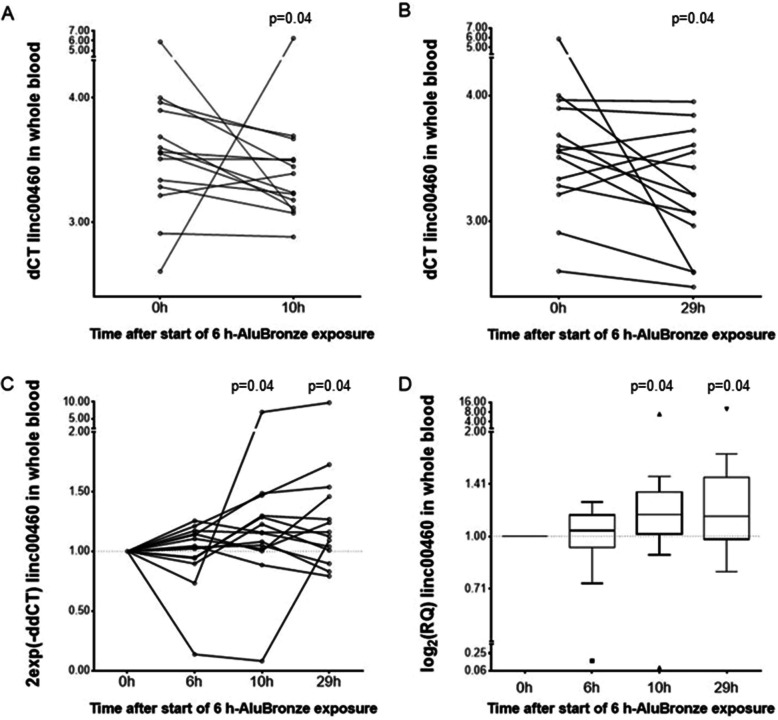
Systemic LINC00460 levels in whole blood cells after controlled short-term metal fume exposure. **A**-**D** Represents alternative depictions of the results. **A**, **B** Paired dot-and-line comparisons of the LINC00460 changes in whole blood cells at 10 h (**A**) and 29 h (**B**) after AluBronze welding fume exposure compared to baseline (*n* = 14). **C** Time course of the 2exp(−ddCT) values of LINC00460 for each participant at the time points. The depicted *P*-values, calculated on the dCT level using the Wilcoxon test, were taken from (**A**) and (**B**). **D** Box-and-Whisker Plot comparison of the log_2_(RQ) values of LINC00460 for each participant at the time points. To enable a symmetric depiction, the y-axis data are presented as antilog to base 2. Calculation of *P*-values as described in (**C**)

The Friedman analysis of variance (ANOVA) was used to examine the LINC00460 dCT levels between points on the metal fume challenge curve (0 h, 6 h, 10 h, and 29 h). A significant overall difference was found (*p* = 0.03). The posthoc analysis using the method given by Conover [34] identified the 10 h and 29 h time points showing weak to moderate significantly increased levels compared to the baseline. The Wilcoxon test confirmed a consistent significant increase of the LINC00460 levels at 10 h (*p* = 0.04; increase in 12 of 14 individuals) (Fig. 2A) and at 29 h (*p* = 0.04; increase in 11 of 14 individuals) (Fig. 2B). The overall time course of the 2exp(−ddCT) values of LINC00460 is shown as a dot-and-line diagram for each participant (Fig. 2C). For visualization, the same data are also depicted as log_2_(RQ) values in the Box-and-Whisker Plot format (Fig. 2D), again showing the moderate but significant increase of LINC00460 levels, especially at 10 h and 29 h.

#### CoroMarker expression levels

**Fig. 3:**
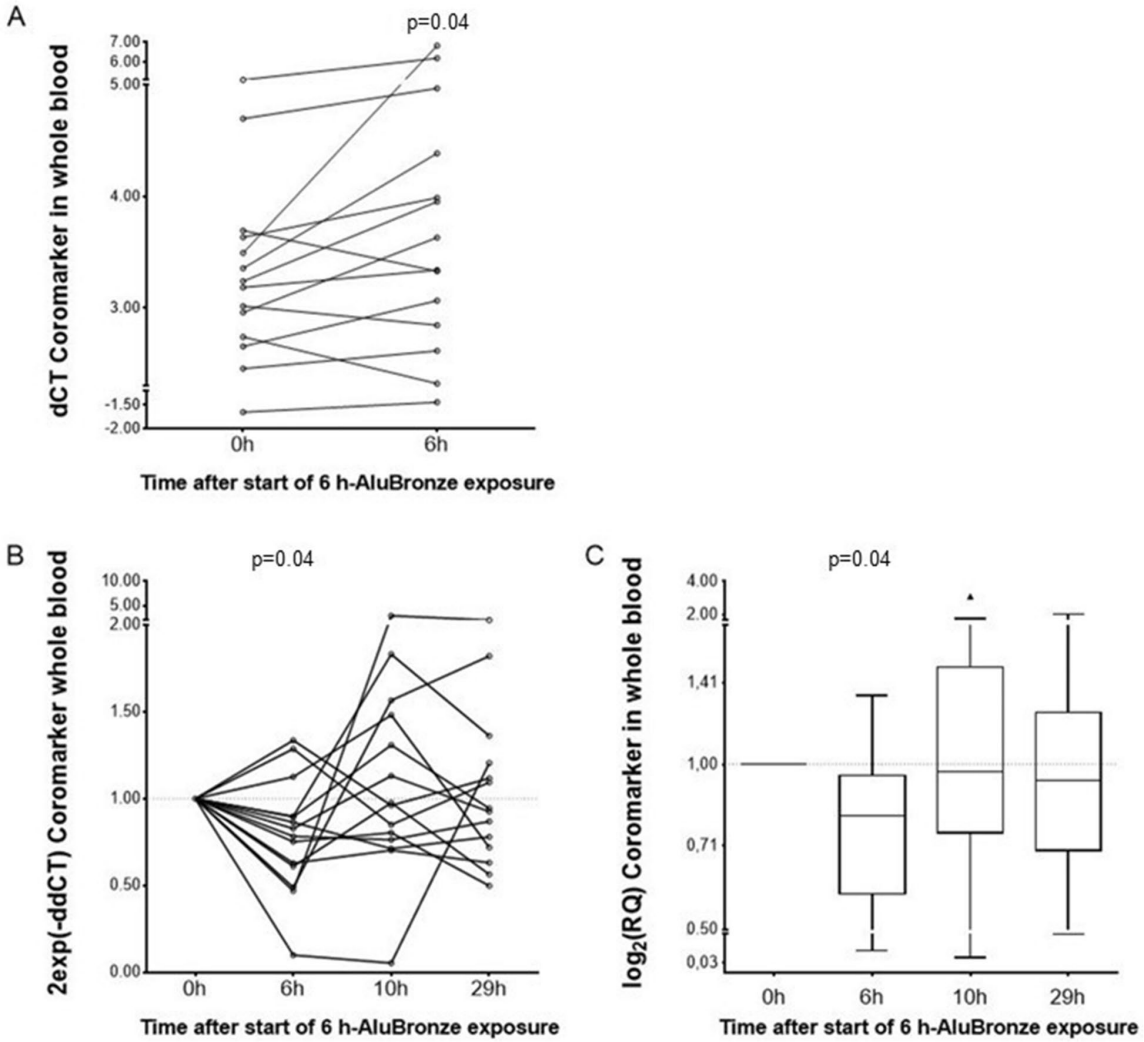
Systemic CoroMarker levels after controlled short-term metal fume exposure. **A**-**C** Represents alternative depictions of the results. **A** Paired dot-and-line comparison of the CoroMarker changes in whole blood cells at 6 h after AluBronze welding fume exposure compared to baseline (*n* = 14). **B** Time course of the 2exp(−ddCT) values of CoroMarker for each participant at the time points. The depicted *P*-value, calculated on the dCT level using the Wilcoxon test, was taken from (**A**). **C** Box-and-Whisker Plot comparison of the log_2_(RQ) values of CoroMarker for each participant at the time points. To enable a symmetric depiction, the y-axis data are presented as antilog to base 2. The calculation of *P*-values as described in (**B**)

The Friedman ANOVA did not show significant differences in the CoroMarker dCT levels between points on the metal fume challenge curve (0 h, 6 h, 10 h, and 29 h) (*p* = 0.34). We nevertheless observed a decrease of CoroMarker levels in 11 of 14 individuals at 6 h compared to baseline. Therefore, we performed the Wilcoxon test to compare the CoroMarker dCT levels at this first time point tested, as in the case of its usage, the later values at 10 h and 29 h (which influenced the Friedman test) would not be needed. We found a significant result (*p* = 0.04) (Fig. 3).

#### MALAT1 and CDR1as expression levels

**Fig. 4:**
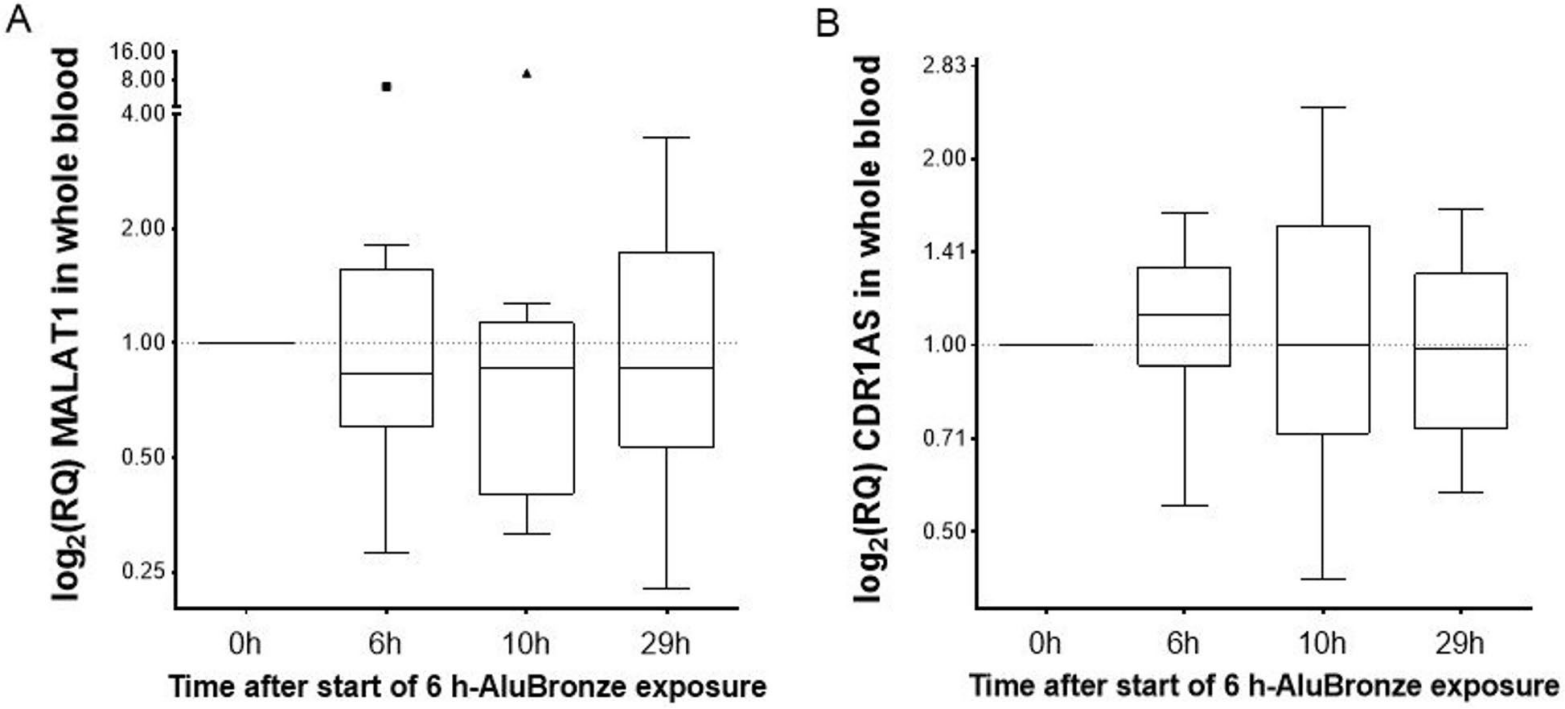
Systemic MALAT1 and CDR1as levels after controlled short-term metal fume exposure. Box-and-Whisker Plot comparison of the log2(RQ) values of MALAT1 (**A**) and CDR1as (**B**) for each participant at the time points. To enable a symmetric depiction, the y-axis data are presented as antilog to base 2

The lncRNA MALAT1 in whole blood cells showed no significant changes after exposure versus before exposure (Fig. 4). The results for CDR1as at 6 h showed a moderate increase in 10 of 14 individuals, although non-significant.

## Discussion

Our study demonstrates, to the best of our knowledge, for the first time, in THP-1 macrophages exposed to zinc and copper metal fume suspensions a significant 2.3-fold upregulation of lncRNA CDR1as at 1 h compared to the unexposed control, as well as a significant 1.4-fold increase of LINC00460 at 2 h followed by a 1.5-fold increase at 4 h. Furthermore, our results show, after controlled short-term exposure of volunteers with Zn/Cu welding fumes, in whole blood cells, a significant downregulation of CoroMarker at 6 h, and a significant upregulation of LINC00460 at 10 h and 29 h.

The non-significant upregulations of CoroMarker in THP-1 macrophages, and of CDR1as in white blood cells, backed up the findings.

The orientation of regulation for LINC00460 and CDR1as were, to a certain extent, comparable to the described in vivo and in vitro settings. However, this was not the case for CoroMarker. Naturally, the in vivo and the in vitro experimental settings differ considerably in terms of the dosage, the nature of the stimulus (original metal fume versus metal fume suspension), and the investigated time points, so comparisons should be made with caution. On the other hand, the data complement each other: Macrophages represent an essential immune cell type in the inflamed lung. And as previously described, according to the computational Multiple-Path Particle Dosimetry (MPPD) model (www.ara.com/mppd), the pulmonary deposition after a 6-hour exposure to the Cu/Zn metal fume is in the range of 0.99–1.22 mg for the particle diameters of 50 nm–250 nm [9]. Moreover, the contact of the macrophages in the lungs occurs earlier than the later systemic influences on the immune cells in the blood.

The concrete chemical/molecular form in which welding fume is present in cell culture and humans should be further investigated in the future. Previous studies by researchers at Karolinska Institute in Stockholm have shown that Cu or Cu/Zn metal particles dissolved in cell culture medium release copper and zinc ions into the cell culture environment [35–37]. A recent study of another welding fume (which also consisted mainly of zinc and copper) showed that its particles mainly consist of CuO and ZnO and that the overall count median diameter (CMD) of the particles was 125 nm [38].

In Zn/Cu-exposed THP-1 macrophages, we observed an increase of LINC00460 at 2 h (*p* = 0.03) and at 4 h (*p* = 0.06) compared to the control. Similarly, we found increases of the whole blood cell LINC00460 in the in vivo setting (compared to exposure start), which were significant among the entire study group and consistent over the later two time points at 10 h and 29 h. According to literature, an increase of the LINC00460 leads to a reduction of mir-149 (in the cell line SUNE-19) [32], and, in turn, the decrease of mir-149 leads to an increase of IL-6 (in cancer-associated fibroblasts in the stroma in gastric cancer) [39]. We previously reported about systemically increased levels of serum IL-6 at 10 h, but not at 29 h, after exposure started in the same study participants [10]. LINC00460 levels were found to be positively associated with cytokines and mediators of inflammation [22]. The functional relevance of the increased LINC00460 levels should be further explored in future studies using different functional model systems, including the silencing or overexpression of LINC00460. In addition, the expression of LINC00460 is increased in several cancers, such as lung adenocarcinoma, and nasopharyngeal carcinoma [32, 40].

We determined a significant downregulation of CoroMarker in whole blood cells from individuals exposed with Zn/Cu welding fume at 6 h after exposure start compared to baseline. This is consistent with the previous report that, after functional enrichment analysis, CoroMarker was clustered with genes negatively associated with inflammation, such as MYD88 [21]. Moreover, blocking of MYD88 has been reported to cause a reduction in IL-6 concentration [41, 42]. Our finding may cause the previously described elevated IL-6 levels in the welding fume exposed individuals at 10 h post-exposure start [10]. However, all these findings and reports are not in line with the following facts: We observed a non-significant increase of CoroMarker at 6 h in THP-1 macrophages exposed to Zn/Cu metal fume compared to control. Consistent with this latter finding, the downregulation of CoroMarker by a siRNA has been shown to decrease proinflammatory cytokine secretion such as IL-6 from THP-1 monocytic cells [21]. CoroMarker is increased in plasma and monocytes from patients with coronary artery disease (CAD) compared to clinical control groups [20, 21]. Functional enrichment analysis showed CoroMarker clustered with genes positively associated with signal transduction, transmembrane transport, and innate immunity [21]. It can be stated that the previous publications about the functions of CoroMarker are somewhat contradictory, which is reflected by our findings, which require further investigation. Moreover, one could aim to also investigate systemic extracellular levels of CoroMarker after more potent inflammatory stimuli at more time points to check whether lower intracellular levels may indicate a previous release of CoroMarker from the immune cells. The unexpected finding of upregulated levels of CoroMarker, similar to LINC00460 and CDR1as, in unexposed THP-1 macrophages after 2 h and 4 h, deserves further investigation. A recent report showed an increase of a specific lncRNA even in resting THP-1 monocyte-derived macrophages [43]. However, the levels of MALAT1 remained unchanged in the same exposure setting.

Our results show a consistent increase of CDR1as in both investigated settings: In Zn/Cu-exposed THP-1 macrophages, we observed a significant distinct increase at 1 h compared to control. And in whole blood cells, we determined an upregulation in 10 of 14 exposed individuals directly after a controlled 6 h exposure compared to baseline. These increases in CDR1as at 1 h or 6 h, respectively, together with the recently described increase of serum IL-6 at 10 h in exposed individuals [10], align with the recent finding that downregulation of CDR1as markedly reduced the IL-6 levels [44]. Moreover, CDR1as was previously reported to be significantly upregulated in the plasma of patients with chronic heart failure [19] and in whole blood samples of patients with an average ischemia time of 200 min after acute myocardial infarction [18]. Considering the increases of the cardiovascular risk markers IL-6, SAA, and CRP after controlled exposure to zinc and/or copper-containing welding fumes [10], our finding of elevated levels of CDR1as adds another potential risk marker for cardiovascular diseases after exposure to Zn/Cu metal particles.

## Conclusion

We investigated the human long noncoding RNAs CoroMarker, MALAT1, CDR1as, and LINC00460 in whole blood of individuals after controlled short-term exposure with ultrafine zinc- and copper-containing metal fume particles under workplace conditions and in human macrophages in vitro. Of note, we found a distinct 2.3-fold increase (*p* = 0.03) of CDR1as, a potential risk marker for cardiovascular diseases, in THP-1 macrophages exposed to Zn/Cu metal fume suspensions for 1 hour in vitro. A non-significant upregulation of CDR1as (*p* = 0.2) could be observed in 10 out of 14 subjects directly after a 6-hour-exposure to Zn/Cu containing welding fumes. Furthermore, we found significant increases of LINC00460, a biomarker associated with elevated IL-6 levels and inflammation, in both the THP-1 macrophage exposure model and the healthy individuals after controlled Zn/Cu exposure. Overall, this provides mechanistic insights and added value to recent studies which describe a putative role of the four investigated lncRNA candidates in the progression and management of inflammatory responses.

## Data Availability

Data are available on request due to privacy or ethical restrictions.

## Appendix 1

### Cell culture and differentiation to macrophages

The THP-1 cells were grown in an incubator at 37 °C and 5% CO_2_ in a humidified atmosphere in RPMI 1640 culture medium with L-glutamine (21,875,091, Thermo Fisher Scientific, Massachusetts, USA), 0.0005% 2-mercaptoethanol (M6250-10ML, Merck KGaA, Darmstadt, Germany), 10% FCS (P30–3702, PAN-Biotech GmbH, Aidenbach) and 1% Penicillin/Streptomycin (15,140,122, Thermo Fisher Scientific, Massachusetts, USA). The THP-1 monocytes were grown as suspension cultures in ThermoScientific Nunc cell culture flasks and split every three to four days with a ratio of 1:10 to maintain a maximal cell titer of 8 × 10^5^ to 1 × 10^6^ cells per ml according to Neubauer chamber cell counts.

Before differentiation, monocytes were adjusted to a cell titer of 5.5 × 10^5^ cells per mL and washed two times with PBS by centrifugation at 300 x g for 5 min. The differentiation of the THP-1 monocytes into macrophages was stimulated by 100 nm of Phorbol-12-myristate-13-acetate (PMA) (Sigma Aldrich, Missouri, USA) in the above-described cell culture medium for 48 h in plastic material from Gibco (Life Technologies, Thermo Fisher Scientific, Massachusetts, USA). The successful differentiation was verified by light microscopy. After differentiation, the cells were cultured in fresh starvation (1% FCS; 1% Pen/Strep; 0.0005% 2-mercaptoethanol) for 24 hours.

## Appendix 2

### Welding fume preparation

The ‘Aachen Workplace Simulation Laboratory’ (AWSL) comprises the emission room, the exposure room, and a ventilation system connecting both rooms, allowing the fume regulation in the exposure room [26, 28]. In the emission room of the AWSL, the welding fume was generated by an experienced welder below a funnel-shaped fume hood for 40 s every 10–20 min. The described welding process is often used for vehicle interiors. It encompasses the MIG (metal inert gas) brazing of galvanized steel with an aluminum bronze (Alubronze) wire (Base metal: hot-dip galvanized steel DC01þZ 275 [1.0330] according to EN 10130; Filler material: CuAl10Fe [Cu6180] according to ISO 24373 in 1.2 mm diameter; GMA process: short arc), as described in detail before [9–11]. The welding fume suspension for the cell culture experiments in vitro was obtained by sonicating 50 mg of the welding fume [26] in 50 ml of starvation medium for 30 min in an ultrasonic bath. Afterwards, a 1:10 dilution series was stored in aliquots at − 20 °C. Thus, the concentration of the welding fume was 100 μg/ml. This stock solution was further diluted at 1:50 using the starvation medium, resulting in a concentration of 2 μg/ml of welding fume in the starvation medium. This diluted solution was later used in the experiment.

## Abbreviations

AWSL: Aachen Workplace Simulation Laboratory’
BAL: Bronchoalveolar lavage
cDNA: Complementary DNA
CRP: C-reactive protein
Cu: Copper
lncRNA: Long noncoding RNAs
MPPD: Multiple-Path Particle Dosimetry
RT-qPCR: Real-time polymerase chain reaction
SAA: Serum amyloid A
SEM: Scanning electron microscope
Zn: Zinc
