# Correction: The human long noncoding RNAs CoroMarker, MALAT1, CDR1as, and LINC00460 in whole blood of individuals after controlled short‑term exposure with ultrafine metal fume particles at workplace conditions, and in human macrophages in vitro

**DOI:** 10.1186/s12995-025-00458-5

**Published:** 2025-05-06

**Authors:** Theresa Scheurer, Jan Steffens, Agnieszka Markert, Miriam Du Marchie Sarvaas, Christoph Roderburg, Lothar Rink, Frank Tacke, Tom Luedde, Thomas Kraus, Ralf Baumann

**Affiliations:** 1 Institute for Occupational, Social and Environmental Medicine, Medical Faculty, University Hospital RWTH Aachen University, Pauwelsstr. 30, Aachen, 52074 Germany; 2 Institute for Translational Medicine (ITM), Medical School Hamburg (MSH), Am Kaiserkai 1, Hamburg, 20457 Germany; 3 Department of Medicine III, Medical Faculty, University Hospital RWTH Aachen University, Pauwelsstr. 30, Aachen, 52074 Germany; 4 Department of Hepatology and Gastroenterology, Charité - Universitätsmedizin Berlin, Augustenburger Platz 1, Berlin, 13353 Germany; 5 Clinic for Gastroenterology, Hepatology and Infectious Diseases, University Hospital Düsseldorf, Moorenstr. 5, Duesseldorf, 40225 Germany; 6 Institute of Immunology, Medical Faculty, University Hospital RWTH Aachen University, Pauwelsstr. 30, Aachen, 52074 Germany

**Correction: J Occup Med Toxicol 17, 15 (2022)**

**https://doi.org/10.1186/s12995-022-00356-0**

Following publication of this article, two errors were identified by the authors.

First, on page 5, the sections ***MALAT1 expression levels*** and ***LINC00460 expression levels*** contain a reversed figure assignment. Specifically, the former refers to Fig. 1D but incorrectly cites it as 1C, while the latter describes Fig. 1C but labels it as 1D.

Additionally, in the Authors’ contributions section, Jan Steffens was accidentally listed as having contributed to the writing of the manuscript.

The incorrect and corrected versions of the relevant part of the Authors’ contributions statement are provided below:

Incorrect

Writing – original draft, Theresa Scheurer and Jan Steffens

Correct

Writing – original draft, Theresa Scheurer.

The original article has been corrected.

